# Cell volume homeostatically controls the rDNA repeat copy number and rRNA synthesis rate in yeast

**DOI:** 10.1371/journal.pgen.1009520

**Published:** 2021-04-07

**Authors:** José E. Pérez-Ortín, Adriana Mena, Marina Barba-Aliaga, Abhyudai Singh, Sebastián Chávez, José García-Martínez

**Affiliations:** 1 Instituto de Biotecnología y Biomedicina (Biotecmed), Universitat de València, Burjassot, Spain; 2 Department of Electrical and Computer Engineering, University of Delaware, Newark, DE, United States of America; 3 Instituto de Biomedicina de Sevilla. Campus Hospital Universitario Virgen del Rocío, Seville, Spain; Ohio State University, UNITED STATES

## Abstract

The adjustment of transcription and translation rates to the changing needs of cells is of utmost importance for their fitness and survival. We have previously shown that the global transcription rate for RNA polymerase II in budding yeast *Saccharomyces cerevisiae* is regulated in relation to cell volume. Total mRNA concentration is constant with cell volume since global RNApol II-dependent nascent transcription rate (nTR) also keeps constant but mRNA stability increases with cell size. In this paper, we focus on the case of rRNA and RNA polymerase I. Contrarily to that found for RNA pol II, we detected that RNA polymerase I nTR increases proportionally to genome copies and cell size in polyploid cells. In haploid mutant cells with larger cell sizes, the rDNA repeat copy number rises. By combining mathematical modeling and experimental work with the large-size *cln3* strain, we observed that the increasing repeat copy number is based on a feedback mechanism in which Sir2 histone deacetylase homeostatically controls the amplification of rDNA repeats in a volume-dependent manner. This amplification is paralleled with an increase in rRNA nTR, which indicates a control of the RNA pol I synthesis rate by cell volume.

## Author summary

Synthesis rates of biological macromolecules should be strictly regulated and adjusted to the changing conditions of cells. The change in volume is one of the commonest variables along individual cell life and also when comparing different cell types. We previously found that cells with asymmetric division, such as budding yeasts, use a compensatory change in the global RNA polymerase II synthesis rate and mRNA decay rate to maintain mRNA homeostasis. In the present study, we address the same issue for the RNA polymerase that makes rRNAs, which are essential components of ribosomes and the most abundant RNAs in the cell. We found that the copy number of the gene encoding 35S rRNA, transcribed by RNA polymerase I, changes proportionally to the cell volume in budding yeast via a feedback mechanism based on the Sir2 histone deacetylase, which guarantees that yeast cells have the appropriate RNA polymerase I synthesis rate required for rRNA homeostasis.

## Introduction

Eukaryotic cells have distributed the transcription work in its nuclei among three different RNA polymerases (RNA pol). RNA pol II transcribes most genes, including those that encode proteins. RNA pol III transcribes some several hundreds of genes (mostly tRNAs and 5S rRNA genes). RNA pol I, specialized in transcribing only one gene, the large rRNA 35-47S gene (depending on the particular eukaryote) is, however, the biggest consumer of ribonucleotides given the need for a vast amount of this precursor of ribosomes [1,2]. Ribosomes are central in protein synthesis machinery and, therefore, ribosome content is a determinant of protein synthesis and, hence, of cell growth and division [1–3]. This implies that given the high metabolic cost of rRNA synthesis, a tightly regulated transcription system is necessary to ensure that the mature rRNA level is coupled with cellular growth demands. In the yeast *Saccharomyces cerevisiae*, RNA pol I uses 60% of all ribonucleotides for its single gene target [1,4]. This extremely high synthesis rate (SR) is possibly due to the high density and speed of this RNA pol [2], as well as the large number of 35S rDNA gene copies. In most eukaryotes, the rDNA gene exists as a long tandem repeat in one chromosome or several [5,6]. So it would appear that the high SR required for the rRNA synthesis needed during active cell growth and proliferation [7] cannot be achieved only by using RNA pol I at its maximum capacity, and the multiplication of the rDNA gene is necessary. This is a slow SR regulation system because the change in the rDNA copy number can be done only during genome replication using an unequal homologous recombination between sister chromatids [6,8,9]. rDNA loci are very dynamic genome regions whose copy number varies considerably in a single species, and even between cells in a single individual [5,10–13]. Apart from regulating the rDNA copy number, regulating rRNA synthesis is also possible by acting on RNA pol I transcription initiation [14] or elongation (see [15] for a recent review), and by controlling the proportion of active rDNA repeats [16]. This last option is feasible because repeats can exist as either silenced chromatin covered by regularly packaged nucleosomal arrays or a transcriptionally active and nucleosome-depleted copy [7,17]. This mechanism is also likely to be slow as the change between chromatin states requires the passage of the replication fork through rRNA genes to reset nucleosome assembly [18].

In order to maintain its identity and physiological features, a cell should keep the concentrations of its molecules constant, or at least within a certain limited range. This homeostatic control involves maintaining the total amount of RNAs and proteins, which are performers of genetic information. Total protein content is homeostatically controlled due to the high protein content of all cells and the high cost of protein synthesis (see [4] for a detailed discussion). This means that the ribosome concentration, and consequently the rRNA concentration ([rRNA]), is strictly controlled because it is a key determinant of protein synthesis (see above). [rRNA] depends on a dynamic equilibrium between its synthesis and decay rates. As stated above, the SR for rRNA is extremely high in actively dividing cells, which means that RNA pol I should be very efficiently controlled to adjust to cell requirements. The rate at which RNA pol I makes the rRNA precursor (also called 35-47S [5]) is known as the nascent transcription rate (nTR, [19]) and accounts for the number of rRNA molecules made by a time unit. As the homeostatic equilibrium depends on concentrations, and not on numbers of molecules, changes in cell volume add another factor that should be taken into account. Thus the SR and the nTR are conceptually different parameters (see ref. [19] for a detailed explanation). The SR accounts for the rate of mature [rRNA] change and varies independently of the nTR because of changes in rRNA maturation and/or in cell volume. By assuming no changes in rRNA post-transcriptional processing, the SR can be calculated from the nTR by dividing it by cell volume [19]. Change in cell volume is a phenomenon linked with not just progression during the cell cycle, but also with cell aging as mother cells undergo progressive size increases across generations [20], and also with the genotype of yeast strains given the influence of many mutations on the cell cycle [21]. When cell volume changes, both the SR and nTR become numerically different because the SR equals the nTR divided by cell volume [19]. However, as the physical parameter that measures RNA pol activity is the nTR, all kinds of regulations act primarily on it.

We have previously shown that the nTR for RNA pol II is regulated differently in cells presenting symmetrical or asymmetrical cell division [22]. In that study we proposed three models or scenarios for nTR regulation depending on the symmetry of division and the involved RNA pol. For RNA pol II in symmetrically dividing cells increases the RNA pol II nTR in parallel with cell volume to keep SR constant. In this scenario (#1), RNA pol II is limiting and the cell volume increase is accompanied by the volume-dependent recruitment of RNA pol II onto target genes, as recently demonstrated in *S*. *pombe* [23]. For budding yeast however, a different scenario (#3) applies: the RNA pol II nTR remains constant in spite of cell volume changes by changing the RNA pol II cell concentration to avoid changes in cell physiology during successive asymmetric cell divisions [22]. Finally, the *S*. *cerevisiae* RNA pol I nTR also remains constant with cell volume, but via a different mechanism: nTR regulation by limiting RNA pol I targets (scenario #2).

In this study, we extend the previous study by focusing on RNA pol I regulation in budding yeast with regards to changes in cell volume. We considered changes in cell volume when comparing the average cell volume in asynchronous actively growing yeast populations. By running different types of experiments to determine the RNA pol I nTR, we found that budding yeast adapts the number of chromosomal rDNA repeats to the population’s average cell volume. We propose a model of regulation, based on that previously proposed by D. Shore [10] and T. Kobayashi [24,25], in which we include the cell volume as the physiological mechanism controlling the Sir2 histone deacetylase activity. This, in turn, regulates the homologous recombination at the rDNA locus and allows the repeat copy number to vary.

## Results

### The nascent transcription rate (nTR) of RNA pol I is proportional to the ploidy of yeast strains and varies with cell volume

In a previous study, which aimed to elucidate the influence of cell size on transcription rates [22], we used non synchronized exponentially growing cultures of yeast strains with different average cell volumes. The set of strains included the wild-type haploid strain BY4741, two haploid mutants (*cln3* and *whi5*) respectively with large and small volumes, and three polyploid (2n, 3n and 4n) strains created by D. Pellman’s group [26] (with cell sizes of approximately 2x, 3x and 4x regarding BY4741). All the strains grew similarly (S1 Table), which excludes any difference in the nTR due to changes in growth rates [27]. We found that the total nTR increased linearly with volume in polyploid cells because of the parallel increase in genome copies. Thus the nTR per genome copy remained constant, and we postulated a scenario for RNA pol I regulation in which the nTR of this RNA pol strictly depended on the abundance of its substrate: 35S rDNA [22].

**Fig 1:**
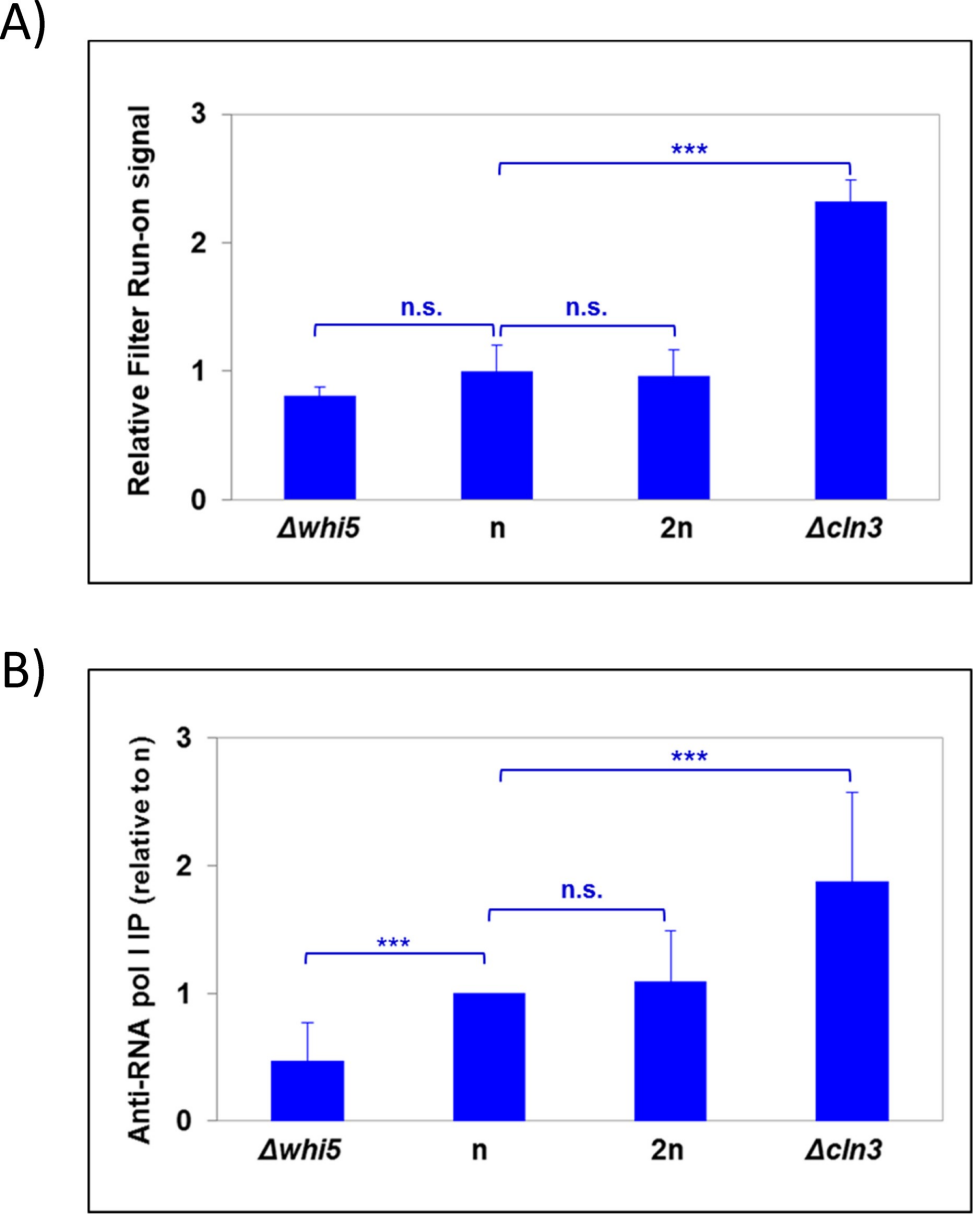
The RNA pol I nTR per genome copy changes in haploid cell size mutants. The nTR was determined in a series of haploid yeast strains with different cell volumes (see S1 Table) and a diploid (2n) control strain for the total RNA pol I nTR by filter run-on (A) that measures the total nTR (I+II+III), or by chromatin immunoprecipitation of its second largest subunit (A135) (B) that measures only RNA pol I nTR. In both cases, the nTR is given per genome copy. A t-test was used for statistical significance: n.s (not significant), ***: p-value < 0.0005.

In this paper, we analyzed the behavior of haploid mutant strains *whi5* and *cln3* by filter run-on (see below) and confirmed it by chromatin immunoprecipitation. Transcriptional run-on is based on capturing elongating RNA pol when rNTPs are depleted by the detergent permeabilization of cells. Then these paused RNA pols are allowed to resume elongation after the addition of external rNTPs, including ^33^P-UTP. It detects all types of nuclear active elongating RNA pols (I, II and III) [28]. The run-on signal is proportional to the number of active elongating RNA pol and allows the nTR between different samples to be compared, provided that the elongating speed of RNA pol does not change. We followed a simplified protocol with the TCA precipitation of the labeled nascent RNA on glass fiber filter discs (“filter run-on”: see M&M). Given that RNA pol II only accounts for 25% of the total, this result informs about RNA pol I+III nTR behavior (Fig 1A). The use of a specific antibody for RNA pol I (against the second largest RNA pol I subunit: A135) confirms that the total nTR per genome copy is much higher in *cln3* and slightly lower in *whi5* than in the wild-type and diploid strains (Fig 1B). Thus, as differences in the nTR cannot be caused by changes in ploidy for haploid mutants of a different cell size, we hypothesized that the variation in the RNA pol I substrate could be due to a change in the number of rDNA repeats.

### The chromosomal rDNA copy number increases in a *cln3* mutant to compensate the bigger cell volume

**Fig 2:**
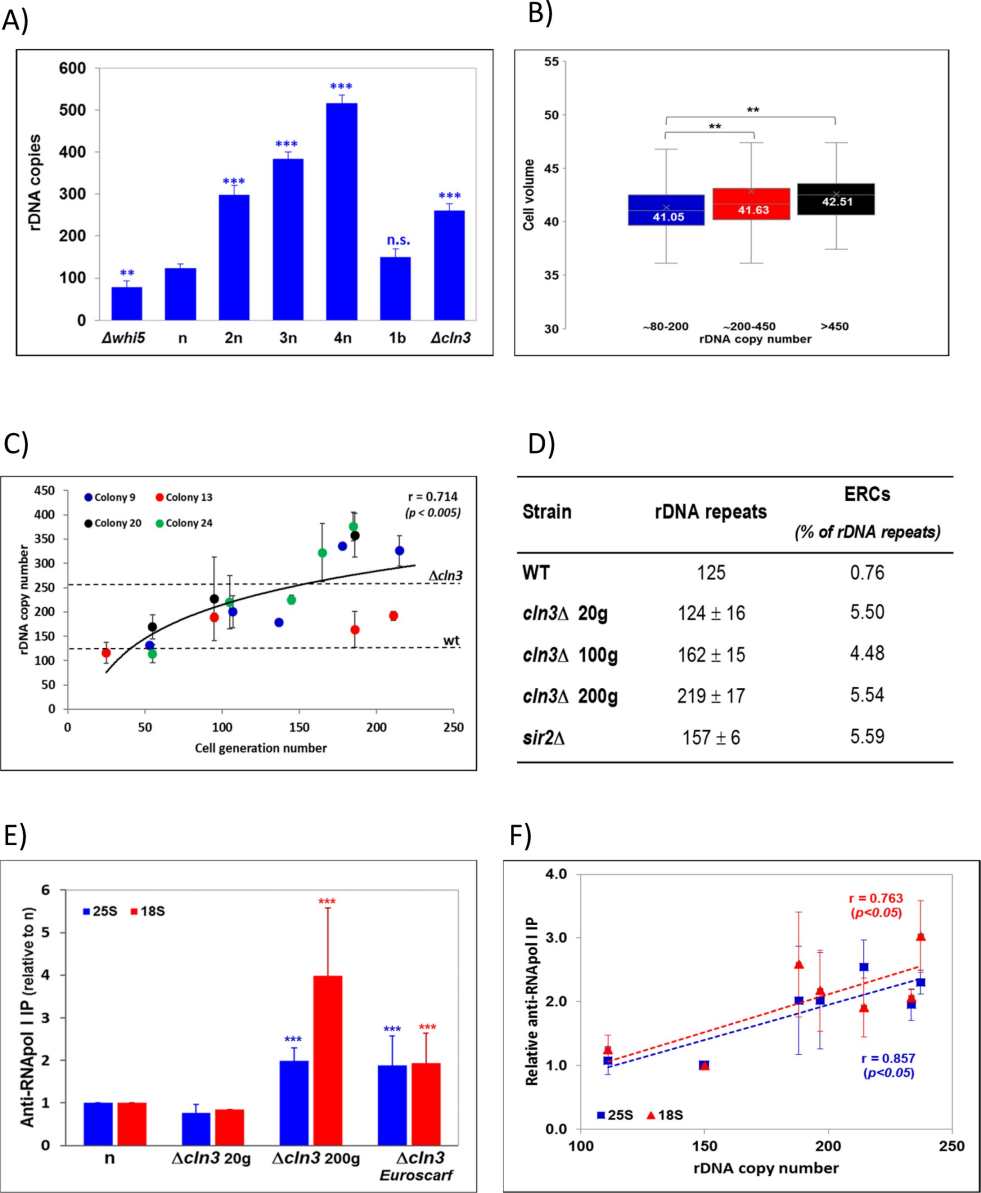
The number of rDNA repeats evolves in a *cln3* mutant to match the optimum value of the RNA pol I nTR required for its cell volume. A) The number of rDNA repeats varies in different strains in such a way that it depends on ploidy, except for cell size haploid mutants (see S1C Fig). A t-test was used for statistical significance: n.s (not significant), ***: p-value < 0.0005; **: p-value < 0.005. B) The average cell volume for the mutant strains from the Kobayashi database [29] was calculated for the three groups of “normal” rDNA repeat copy number (#80–200), high copy number (#299–400) and very high copy number (>450) using the cell volume data (in fL) from [21]. A similar plot, but using the cell volume data (in arbitrary units) from [32], is shown in S1B Fig. The difference was maintained even with the mutants with a very low growth rate (using the data from [32]). We filtered out mutants at the 1.3-, 1.5- or 1.7-fold lower growth rates than the wild type. The values obtained for average cell volumes were exactly the same as those shown in panel B. C) In an “early” *cln3* mutant, the copy number is small, similar to its parental wt haploid (n: BY4741) when it is a recent transformant, but increases along with the number of generations for four independent clones (#9,13,20,24). The dashed horizontal lines mark the repeat copy number for strains Euroscarf *Δcln3* (BQS2006) and wt (BY4741). D) Extrachromosomal rDNA circles (ERCs) analysis. The DNA from different strains was isolated and quantified, electrophoresed, blotted and hybridized with ACT1 and 18S rDNA probes. See S2 Fig for an example of electrophoresis. The number of repeats corresponded to the 18S/ACT1 ratio and relativized to the wild type (125 copies). The percentage of ERCs corresponded to the ratio of all the ERCs bands (see S2 Fig) to the genomic DNA band. Two repeats of the experiment were done. The values represent the averages and standard deviations (SD) for them. The wild-type sample (BY4741) had no SD because it was taken as an internal reference of intensity in each replicate using the calculated copy number of 125 repeats (panel A). E) The RNA pol I nTR of the different *cln3* mutants varies according to the rDNA repeat number, as determined by the chromatin immunoprecipitation of the second largest RNA pol I subunit (A135) onto either the 25S (blue) or 18S (red) transcribed regions. Strain *Δcln3* Euroscarf is BQS2006 (see S1 Table). A t-test was used for statistical significance of the differences between the freshly obtained (20 generations, 20g) ***Δ****cln3* mutant and the evolved ***Δ****cln3* (“late” 200g and Euroscarf) mutants: ***: p-value < 0.0005. F) The RNA pol I nTR of the *Δcln3* mutants increase in parallel to the rDNA repeat number during the evolution experiment. The nTR was calculated as in E).

The rDNA gene in *S*. *cerevisiae* forms tandem repeats between 100–200 copies [9]. The repeat copy number has been shown to vary between different mutants [29]. It is also known that rDNA repeats can pop-out, which leads to the production of extrachromosomal rDNA circles [30]. ERC production can also be a mechanism of rDNA amplification [31]. Therefore, we hypothesized that the number of rDNA repeats increased in the *cln3* mutant and decreased in *whi5* to adjust its number to the average cell size. To test this, we measured the rDNA repeat number in the series of yeast strains by qPCR. Figs 2A and S1A show that the total copy number in relation to a single copy gene control (*ACT1*) is more than double in the *cln3* mutant and slightly lower in the *whi5* mutant compared to BY4741. In fact the relative rDNA copy number change in both mutants with regards to BY4741 (2.2 and 0.9) comes very close to the relative changes in the observed RNA pol I nTR (2.3 and 0.85, from the Filter run-on data in Fig 1A). In order to check if the copy number of rDNA repeats is related to cell volume, we checked the Yeast rDNA Stability (YRS) database of Kobayashi’s group in which about 4800 yeast gene knockout strains are classified as four groups according to the number of rDNA repeats [29]. We compared the average cell volume (taken from [21] and [32]). In Figs 2B and S1B we can see that cell size significantly increases in the cells with a bigger rDNA copy number. Given that the genetic determinants of cell size are multiple [33], the small, but significant, correlation demonstrates that the number of rDNA repeats tends to increase with cell size irrespectively of the physiological reason for cell increase.

If the mutants had a different repeat copy number from the wild-type strain that they derive from, we hypothesized that it would be due to a secondary effect after deleting the target gene. This is because the cell, where deletion occurs, has a wild-type number of rDNA repeats. We hypothesized that the rDNA copy number would be adjusted after gene deletion by successive steps across genome replications [34]. To test this, we ran a 200-generation culture of four freshly obtained (“early”, <20 generations) ***Δ****cln3* mutants. We first checked the cell size of these mutants and found that it was indistinguishable from a “late” *cln3* mutant obtained from the Euroscarf collection in which we performed previous experiments: 83±10 fL (S1C Fig, S1 Table). We also checked that these four mutants had a wild-type rDNA copy number. Then we cultured these ***Δ****cln3* mutants in YPD for approximately 200 generations by refreshing cultures daily in new medium every 10–12 generations. Fig 2C illustrates that all the *cln3* strains progressively increased the number of rDNA repeats to up 250 on average, although variability among strains was wide. Then we checked if the copy number increase was due to ERC amplification. ERCs can be distinguished from chromosomal rDNA copies by agarose electrophoresis and Southern blotting because they migrate as separate multimeric bands given their circular and supercoiled nature [30,31]. In Fig 2D it can be seen that *cln3* strains have a higher level of ERCs than the BY4741 wild type, and within the range of the *sir2* mutant, but that this level did not increase during the evolution from an “early” mutant to a “late” one. Thus, it would seem that a fresh *S*. *cerevisiae cln3* mutant had an intrinsic enlarged size because of its longer G1 period [29,35], and not as a result of an altered rDNA copy number. Its larger size can provoke a defect in the effective SR of 35S rRNA if the nTR was not corrected. The plasticity of the rDNA locus, however, may lead the chromosomal repeat copy number to rise and, consequently, the nTR. In fact the measurements of elongating RNA pol I by immunoprecipitation with Anti-A135 Ab confirmed that the actual RNA pol I nTR in the 200-generation-old *Δcln3* cells was higher than in the <20-generation-old one (Fig 2E), and that it increased in the newly generated ones across generations during the evolution experiment in parallel to the increase in the rDNA repeat copy number (Fig 2F).

### A model for RNA pol I transcription regulation by cell volume

**Fig 3:**
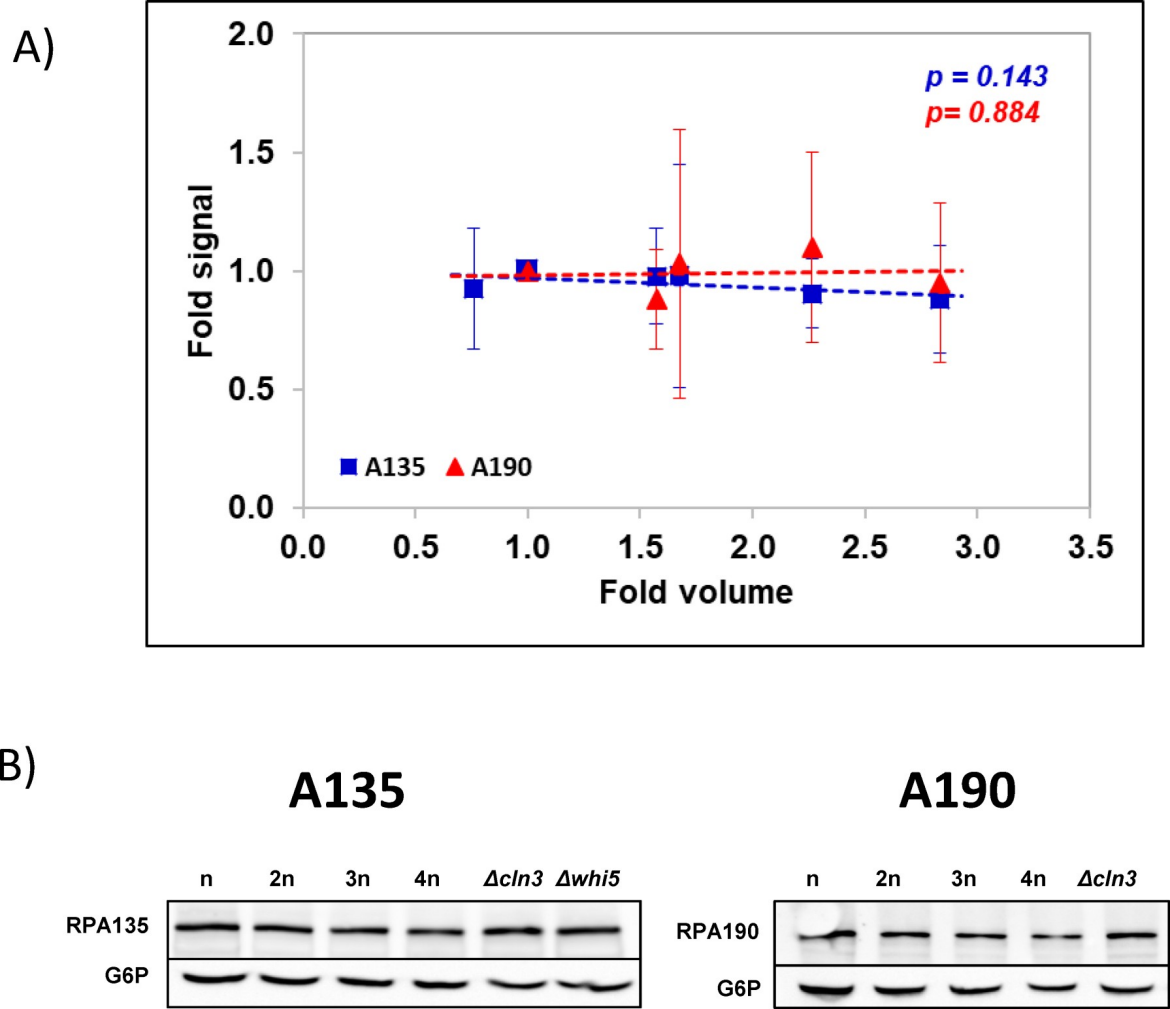
The cellular concentration of RNA pol I does not vary with cell volume. A) The RNA pol I concentration in the different polyploids (n-4n) and haploid mutants (*whi5*, *cln3*) shown in Fig 2A was calculated by Western blot using approximately the same amount of total protein and correcting by the G6P-DH signal. An antibody against the second largest RNA pol I subunit (A135, blue squares) and one against the largest RNA pol I subunit (A190, red triangles) are shown. The graphs (representing the fold change regarding the haploid strain) represent the average and standard deviations of three biological replicates. B) In both cases, an example of a Western blot is shown below. The slopes of the trend lines in panel A) do not significantly deviate from the horizontal according to an F-test (p-values are shown).

We previously published a model for RNA pol I transcription regulation with cell volume, which is based on large excess and a high affinity of this RNA pol to its targets (scenario #2 in [22]). This model predicts that [RNA pol I] does not decrease with cell volume to keep the nTR constant (unlike RNA pol II, see [22]). We used the set of yeast strains with different cell volumes (see S1C Fig) for the Western blot quantification of the two main subunits of this RNA pol to find that the RNA pol I concentration remained constant (Fig 3).

**Fig 4:**
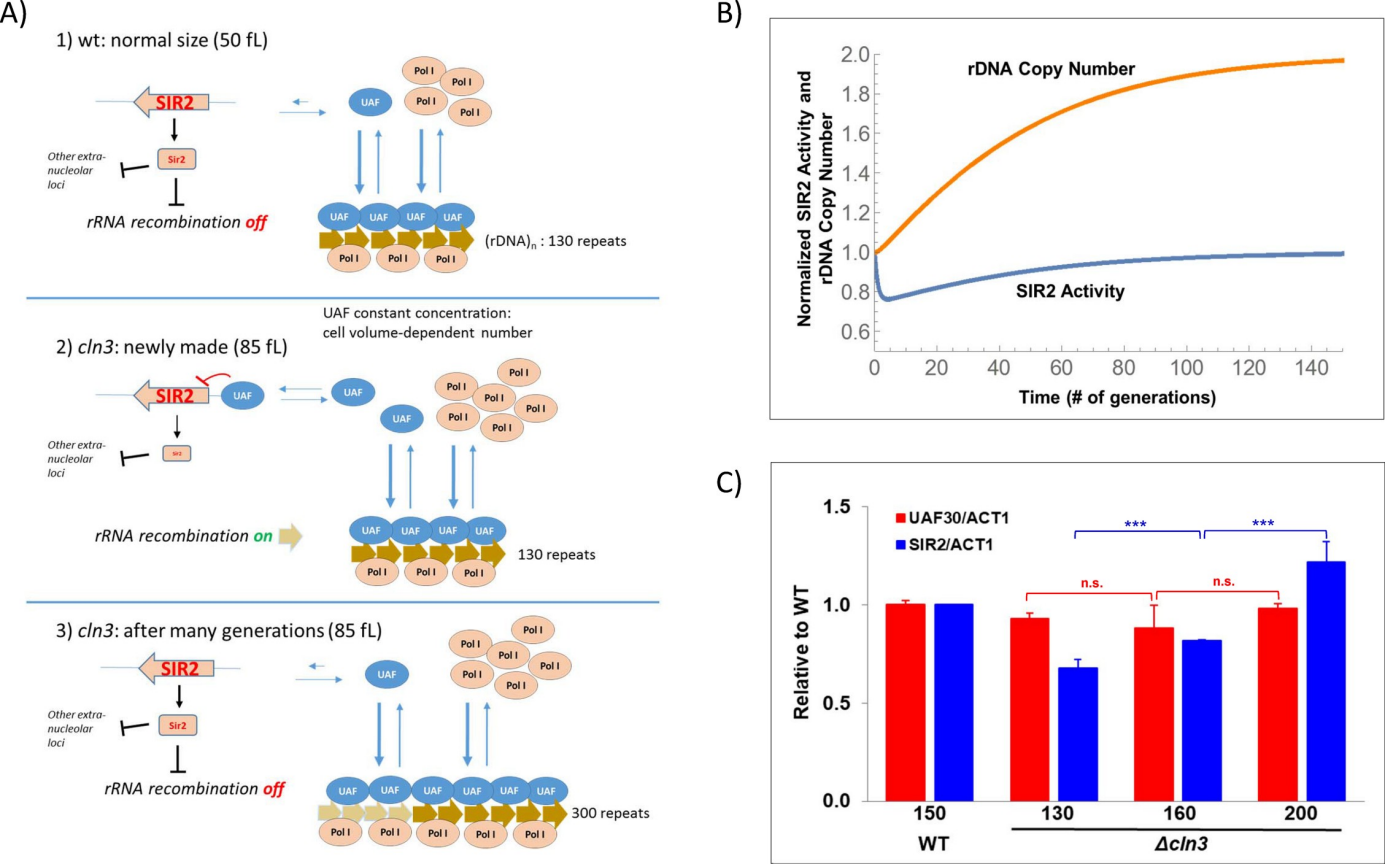
Model for rDNA copy number regulation by cell volume. A) A cartoon model for cell size control on the rDNA repeat copy number is shown (see the main text for details). B) A mathematical model (see S1 Appendix) predicts that the Sir2 activity (blue line) suddenly decreases after cell enlargement in cell that becomes *Δcln3* and that progressively recovers in parallel to the increase in rDNA copy number that results from the increased recombination at the rDNA locus and the selection for higher copy number successive generations. C) The relative levels of *UAF30* and *SIR2* to a reference gene (*ACT1*) in the *cln3* strains of different numbers of generations and repeats (130, 150, 200) during the evolution experiment determined by RT-qPCR are shown. Bars are relative to the value a wild type (strain 1b S1 Table, WT, 150 repeats) used as a reference. The x axes reveal the rDNA copy number of the different strains used. A t-test was used for statistical significance: n.s. (not significant), ***: p-value < 0.0005.

In that scenario [22], the total nTR was, thus, driven by the actual number of rDNA gene copies present in each strain. For the previously described 200-generation-evolved *cln3* mutants, this would appear to be modulated by an amplification of the rDNA repeats occurring along many genome replications. T. Kobayashi’s group has shown that the rDNA locus is very plastic and undergoes expansions and contractions. Expansions are driven by unequal sister-chromatid recombination, which produces two cells with different repeat copy numbers, followed by a gradual evolution (see [34,36]). This evolutionary pathway fits our previous results with *cln3* mutants. A recent paper [24] postulates that the control of homologous recombination is due to the action of histone deacetylase Sir2 which, in turn, is controlled at the transcription initiation level by the upstream activator factor (UAF) complex. The UAF is both an activator of RNA pol I transcription and a repressor of *SIR2* transcription by RNA pol II. Here we adapt this model to account for our observations as regards cell volume. Our model (Fig 4A) assumes that both the UAF and RNA pol I have very high affinity constants for the rDNA promoter. Under steady-state growth conditions, both proteins saturate the available (open chromatin) repeat copies. Under steady conditions, the UAF is in a limiting amount [24], whereas RNA pol I comes in excess [7,22]. When the *CLN3* gene is deleted after transformation, the wild-type cell instantaneously becomes a *Δcln3* mutant and changes its cell cycle to become larger than the wild type. As most proteins maintain their concentration independently of cell volume, the number of the UAF and RNA pol I molecules/cell increases proportionally to cell volume. This has no effect on RNA pol I behavior because the considerable molar excess over its targets renders the change in the number of free molecules irrelevant. Conversely for the UAF, the proportional change in the number of molecules to the increase in volume, because of the constant rDNA copy number, provokes a change in the free [UAF]. UAF also has affinity for the *SIR2* promoter, albeit much lower than for rDNA [24]. Therefore, an increased concentration of free UAF leads to *SIR2* gene repression. Thus in larger cells, the UAF will repress *SIR2* transcription which, in turn, will cause the de-repression of the homologous recombination and will promote an increased copy number [34]. When the number of rDNA repeats reaches the necessary number to sequester most of the free UAF molecules present in the new cell volume, the system will return to the steady state.

We studied this homeostatic control of the rDNA repeat copy number using a differential equation-based mathematical model (S1 Appendix). Intriguingly, our mathematical model corresponded to an integral feedback mechanism to control the concentration of rDNA repeats at a prescribed set point. Such integral feedbacks have recently been shown to be essential for robust perfect adaptation when faced with environmental perturbations [37]. We refer readers to the Supplementary Information for mathematical modeling details. It can be seen that the model predicts a fast decrease in Sir2 activity after cell volume increase followed by a recovery to a normal levels in parallel to the increase in rDNA copy number (Fig 4B) similar to the increase in rDNA repeats observed in the *Δcln3* mutant evolutionary experiment (Fig 2C).

A prediction of this model is that the total cell [UAF] will be always constant, but excess free [UAF] will repress *SIR2* expression in freshly made *cln3* mutants and will recover to its wild-type levels after 200 generations. Fig 4C shows that the *UAF30* subunit mRNA expression is constant in the *cln3* strains with a different rDNA copy number, but *SIR2* expression is lower in the *cln3* strains with a small rDNA copy number and increases to wild-type levels in the strains with a larger rDNA copy number. The observed profile of *SIR2* mRNA fits to that predicted for Sir2 activity by the mathematical model (Fig 4B).

## Discussion

How the cell controls the activity of its RNA polymerases (i.e. their nTR) to obtain the right synthesis rate depends on cell division symmetry [22]. Symmetrically dividing cells, such as human fibroblasts and *S*. *pombe*, increase the number of elongating RNA pol II by keeping its cellular concentration constant independently on cell volume, while maintaining a very strong association constant with gene promoters [38,39]. In this way the equilibrium is strongly displaced toward the chromatin binding of RNA pol II, which assures that the increase in RNA pol II molecules with cell volume, required to maintain the concentration constant, will directly increase the nTR. We called this “scenario #1” in our previous study [22]. It has been recently demonstrated in *S*. *pombe* that the RNA pol II amount scales with cell volume during the cell cycle and has a direct consequence on transcription initiation rates [23]. Due to the asymmetric division in budding yeast, the nTR cannot increase with cell volume because it would provoke a never-ending increase in daughter cells with every generation [22]. Instead we found that *S*. *cerevisiae* lowered the SR through volume increase because the RNA pol II concentration was down-regulated by cell volume and mRNA homeostasis was maintained by a compensatory increase in global mRNA stability [22].

The conceptual problem solved for RNA pol II in different organisms is applicable to the other nuclear RNA pol. Our previous study suggested that in budding yeast, RNA pol I had also a constant nTR per gene copy due to an excess amount of this RNA pol regarding its rDNA template (which is limiting). We proposed a new scenario (#2), in which the rise in the nTR in large-sized polyploid cells would be caused by their increase in ploidy, which would proportionally augment the total number of rDNA repeats by keeping the SR constant. However, we found that other yeast strains with smaller or bigger sizes than the wild type, but with haploid genomes (e.g. *cln3* and *whi5*), also adapted their total nTR to their cell size. We suggested that it could be related to the plasticity of rDNA repeats, which are known to be variable in number [29]. In the present study, we performed a series of experiments that confirmed our hypotheses: the nTR per gene copy was constant in asynchronous populations of yeast strains with different average cell volume. Thus, the total nTR could increase with cell volume by increasing the rDNA copy number with ploidy increase [22] or by altering the rDNA repeats per genome (this paper). We also found that the number of rDNA repeats in budding yeast adjusted to the required RNA pol I SR by a mechanism based on the transcriptional control of the histone deacetylase *SIR2* gene (Fig 4). Our model of rDNA copy number control by cell volume is based on T. Kobayashi’s previous model [24,25] called “Musical chair”. This model predicts that, similarly to the scenarios proposed for RNA pol II transcription rate control (see above), a limiting amount of a DNA binding protein (UAF), together with its high binding constant to the rDNA promoter, would lead the concentration of free UAF to be negligible in wild-type cells, but would be exquisitely dependent on the number of rDNA repeats. Iida and Kobayashi [24] demonstrated that the UAF is also able to bind the *SIR2* gene promoter, albeit with a much lower binding constant than for an rDNA promoter, by acting as a repressor. In this way, Sir2 histone deacetylase activity becomes dependent on the number of free UAF molecules. The silencing activity of Sir2 on an ncRNA gene placed within the rDNA repeat is necessary to repress the homologous recombination between rDNA repeats during replication, which is the origin of repeat copy number variability [6,23,24]. This variability would then allow the evolution of the appropriate rDNA copy number for its genetic features and cell volume. Interestingly, the increase in the rDNA copy number that happens in cells with a few repeats has been demonstrated to depend on the target of rapamycin (TOR) pathway [40]. Only when TOR is active can rDNA amplification occur. This amplification also takes place, as in our study, by increasing chromosomal copies, and not by ERCs increase. Our studies have been done in exponential growth-rich media when TOR is active. The evolution experiment, however, included the daily re-inoculation of post-diauxic cultures to simulate common yeast growth conditions. As most cell divisions occur in the exponential phase during the time periods when TOR is active, this has no major effect on our evolution experiment and in similar ones performed by other authors under comparable conditions [36,40]. The TOR pathway is also a positive effector on the transcription of ribosome proteins genes by acting via Sfp1 and Sch9 activators and Fhl1 and Ifh1 transcription factors [41]. Our previous results established that ribosome protein expression is regulated by cell volume at the transcription and mRNA stability levels [32]. Our present results and those previously obtained in Houseley’s lab [40] show that TOR acts on the RNA pol I transcription level by controlling, through Sir2 and other histone deacetylases, the rDNA repeat number. These results are added to the previously known effect of TOR regulation of RNA pol I transcription at the initiation level [14]. The necessary parallel mechanism to coordinate rRNA levels with ribosome protein synthesis, thus, acts in *S*. *cerevisiae* differently on mRNAs, which can be easily regulated at the stability level, and rRNA, which is much stable, but can be regulated at the gene copy level.

Regarding the mechanism that controls rDNA amplification we hypothesized that any change in cell volume with no change in rDNA repeats would alter the concentration of free UAF molecules. This occurs because if UAF expression does not change (constant cellular protein concentration), the increase in cell volume involves a parallel increase in the total UAF molecules per cell but, because of the invariant rDNA target number, the concentration of free UAF will increase and lead to *SIR2* transcription repression. Then we found that the increase in rDNA copies which occurred in a freshly made *cln3* mutant (Fig 2C), with a wild-type copy number, but a much larger cell size (S1C Fig), provoked a decreased *SIR2* expression, which recovered and reached the wild-type values (Fig 4C) along with the progressive increase in rDNA repeats seen across successive generations (Fig 2C). This change took place without any changes in UAF30 expression (Fig 4C). Interestingly, a previous study found that *SIR2* expression was reduced in large cells and increased in small wild-type cells, although it was not related to UAF regulation [42]. The increase in rDNA can also happen by the generation of extrachromosomal minicircles (ERCs). This has been demonstrated to occur for balancing the number of chromosomal repeats [31]. In that study, however, ERCs were generated when the number of repeats lowered. In contrast, in our study the proportion of ERCs did not change, in spite of chromosomal rDNA amplification. Therefore, the increased number of rDNA copies was not due to a greater accumulation of ERCs (Fig 2D).

Previous studies have shown that rRNA synthesis is determined by the RNA pol I loading rate and not by the number of active genes, and that the number of elongating RNA pol I molecules remains constant in spite of the change in rDNA repeats [16]. This result, however, was found for cases in which the number of rDNA repeats was very small (42 repeats). In that case, the nTR increased because of the increase in both the proportion of active repeats and RNA pol I density. In our case, however, the number of active RNA pol I molecules varied proportionally to the rDNA copy number (Fig 2F), which supports the previous suggestion made by Houseley [40], which indicates that regulation by gene copy number can be a safer strategy and one that does not provoke genome instability in the long term (discussed in [40]).

We cannot rule out that the changes in the proportion of active genes and/or kinetic parameters of RNA pol I happen also in mutants *whi5* and *cln3*, but the parallel increase in rDNA repeats (Fig 2A) and the number of elongating RNA pol I molecules (Figs 1 and 2F) suggest that this is the simplest explanation. The different increase observed for several *cln3* transformants (Fig 2C) may, however, indicate that additional modes of regulation for the RNA pol I nTR with cell volume could exist.

Finally, another question to arise from our model is the possible additional effects that the change in Sir2 activity might have on other cellular processes. This histone deacetylase is responsible for maintaining chromatin silencing in telomeric regions, and for mating type loci and rDNA [36]. At rDNA, Sir2 acts in a complex called RENT, which interacts with the 35S promoter. Although Sir2 seems dispensable for RNA pol I transcription, its tethering to the 35S promoter seems important to strike a balance between Sir2 action in rDNA and at telomeres (see [36] for a detailed description). Any shrinkage in the rDNA array not only releases Sir2, but also causes both an increase in telomeric and mating-type gene silencing and a proportional down-regulation of the *SIR2* gene to repeat loss [10]. The down-regulation of Sir2 can be explained by the UAF repression described in the Musical Chair model [25]. D. Shore’s group proposed that there is an equilibrium between the nucleolar and non-nucleolar pools of the Sir2 protein, and that rDNA “buffers” the amount of Sir2 in a cell [10]. Thus, cell volume changes could also have effects on telomere and mating-type silencing.

## Materials and methods

### Yeast strains, media and growth conditions

The *S*. *cerevisiae* strains used herein are summarized in S1 Table. The *S*. *cerevisiae* cells were grown in either liquid YPD (2% glucose, 2% peptone, 1% yeast extract) or YPGal (2% galactose, 2% peptone, 1% yeast extract) media. Experimental assays were performed with the cells exponentially grown for at least seven generations until OD_600_ ~0.4–0.5 at 30°C.

The growth rate (GR) was calculated by growing 50 mL of yeast cultures in 250 mL flasks with shaking (190 rpm) at 30°C. Aliquots were taken every 60 min in the exponential phase and their OD_600_ were measured. GRs (h^-1^) were calculated from growth curves in YPD and/or YPGal and are shown in S1 Table.

### *cln3* strain evolution experiment

For culture evolution purposes, the *Δcln3* mutants were made from strain BY4741 by substituting the natural allele for the KanMX4 PCR-amplified cassette with the oligos described in S2 Table following the lithium acetate protocol [43]. An rDNA copy number alteration due to Li^+^ was discarded by qPCR test (see below) in the obtained clones. Small colonies (1 mm diameter) were assumed to contain ~2×10^5^ cells and corresponded to progenies after about 18 generations, starting from single ancestor cells [34]. The subcultures in liquid YPD medium were started and growth was followed by measuring OD_600_. Repeated subculturing was done by inoculating up to 10^4^ cells/mL into 20 mL of fresh medium and allowing them to grow for 24 h to 5–6 OD_600_ (to about 10^8^ cells/mL, 10–11 generations). An aliquot of the culture was spun down daily, the supernatant was discarded and the cell pellet was frozen onto liquid nitrogen. Four different small colonies *Δcln3* (#9, 13, 20, 24) were used to make four replicates of the experiment.

### Cell volume determination

The median values of the cell volumes of the population were obtained by a Coulter-Counter Z series device (Beckman Coulter, USA), as previously described [22]. The absolute values (in fL) are shown in S1 Table.

### RNA extraction, RT-qPCR for mRNA levels

To determine the amount of RNA, cells were grown in rich media until the exponential phase (see above). Then total RNA was purified by phenol:chloroform extraction, as described in [44] in three biological replicates and was quantified by an OD_260_ estimation in a Nanodrop device (ThermoFisher Scientific) or using the Qubit RNA BR Assay kit (Thermo Fisher Scientific) measured in a Qubit fluorimeter for accurate quantification purposes.

The expression of *SIR2* and *UAF30* was measured by RT-qPCR in the DNase I-treated RNA samples, extracted as previously described before being normalized against the *ACT1* mRNA levels. Specific primers were designed for this aim and are listed in S2 Table. The reverse transcription of mRNA was carried out using an oligo d(T)_15_VN with Maxima Reverse Transcriptase (Thermo Fisher Scientific). cDNA was labeled with SYBR Pre-mix Ex Taq (Tli RNase H Plus, from Takara) and the Cq values were obtained from the CFX96 Touch Real-Time PCR Detection System (BioRad).

### Determination of the nascent transcription and synthesis rates by filter run-on

For the total (RNA pol I + II + III) nascent transcription rates (nTR), a run-on experiment was used, followed by TCA precipitation onto a glass fiber filter. For these experiments, the *S*. *cerevisiae* cells were grown to OD_600_ 0.4–0.6. Three biological replicates were performed for each yeast strain. For each sample, two aliquots of 2.5x10^7^ cells were collected by centrifugation at 4,000 rpm for 3 min in a 1.5 mL Eppendorf tube. Pellets were resuspended in 0.5 mL of 0.5% sarkosyl and spun down again by centrifugation under the same conditions as above. Supernatants were discarded to completely eliminate sarkosyl. Then the pellet was resuspended in 7.2 μL of distilled water. The run-on pulse was performed by adding to the cell pellet, per sample, 9.87 μL of a master transcription mix, composed, per sample, of 7.5 μL of 2.5× transcription buffer (50 mM Tris-HCl, pH 7.7, 500 mM KCl, 80 mM MgCl2), 1 μL of the rNTP mix (10 mM each ATP, CTP and GTP), 0.375 μL of 0.1 M DTT, 1 μL of UTP (0.9 μL of 3 μM cold UTP and 0.1 μL of 3 μM [α-^33^P] UTP, Perkin Elmer, 3000 Ci /mmol, 10 μCi/μL) at a final, per sample, volume of 18.75 μL. To allow transcription elongation, the mix was incubated with agitation (650 rpm) for 5 min at 30°C. The reaction was stopped by adding 82 μL of cold distilled water to the mix and leaving it on ice. To measure the total amount of radioactivity present in the mix (‘Total’), 15 μL of the reaction were directly spotted onto glass fiber paper discs and dried in an aerated heater at 65°C. Another 15 μL aliquot of the mix was spotted onto glass fiber discs and precipitated by soaking it in 4 mL of 10% (v/v) of trichloroacetic acid (TCA) at 4°C for 20 min. TCA was removed and a new wash of 4 mL of cold TCA (10% v/v) was added for 10 min. TCA was removed and discs were washed with 3 mL of cold 70% (v/v) EtOH. Discs were dried again in a heater at 65°C. Once dried, 3.5 mL of a scintillation cocktail (Normascint #22, Scharlau) were added to each disc for radioactive counting purposes. All three biological replicates were performed in three technical replicates for TCA and two for the total and were averaged. For each individual sample, incorporation was calculated as “TCA/Total” being the total nTR. Synthesis rates (SR) were estimated by dividing the nTR values by cell volume (see above). The nTR values per genome copy were obtained by dividing the nTR by ploidy.

### Determination of the nascent transcription and synthesis rates by chromatin immunoprecipitation of RNA pol I

As an alternative way to measure the nTR, the recruitment of RNA polymerase I to rDNA chromatin was assayed by a chromatin immunoprecipitation (ChIP) analysis with a specific antibody for RNA pol I. Cells were grown in rich medium until the exponential phase. Crosslinking between proteins and the associated DNA was performed by adding formaldehyde to 0.75% for 15 minutes. Samples were sonicated 12 times (30 sec on, 30 sec off) at high intensity in a Bioruptor (Diagenode) device. Chromatin fragments were immunoprecipitated using a rabbit polyclonal antibody (diluted 1:50) against RNA polymerase I subunit 135 (A135), coupled to protein A-conjugated magnetic beads (Thermo Fisher Scientific). DNA fragments were then amplified by qPCR and the Cq values were analyzed. SYBR Pre-mix Ex Taq (Takara) was used for qPCR following the manufacturer’s instructions. The reaction was performed in the CFX96 Touch Real-Time PCR Detection System (BioRad). The primers designed to detect ribosomal genes (25S, 18S) are listed in S2 Table. Their localization on the rDNA repeat is shown in S1D Fig. Signals were normalized against the *ACT1* gene signal to give the nTR values per genome copy.

### qPCR quantification of rDNA repeats

The estimation of the number of ribosomal RNA gene (rDNA) repeats was measured by qPCR and normalized against the *ACT1* gene signal. For this purpose, genomic DNA was purified by a standard phenol:chloroform extraction [45] and precipitated with ethanol. Primers were designed against a specific region in the rDNA cluster (5.8S) and are listed in S2 Table. DNA was labeled with SYBR Pre-mix Ex Taq (Tli RNase H Plus) from Takara and the Cq values were obtained from the CFX96 Touch Real-Time PCR Detection System (BioRad).

### Western blot analysis

Protein extraction from 1-2x10^8^ yeast cells was done by suspending the cell pellet in 200 μL of NaOH 0.2M. After 5 minutes, samples were centrifuged and then mixed with 100 μL 2X SDS-PAGE loading buffer. They were incubated at 95°C for 5 minutes to completely extract proteins from cells. For the Western blot analysis [46], similar protein amounts were injected into SDS-PAGE acrylamide gels under reducing and denaturing conditions. Rabbit polyclonal antibodies against A190 and A135 RNA pol I subunits were assayed (diluted 1:5000) in this study, which were provided from O. Calvo and C. Fernández-Tornero. Glucose 6-phosphate dehydrogenase (G-6-PDH) was used as an internal control by re-incubating the same blots with a specific antibody (A9521 from Sigma Aldrich). Bound antibodies were detected using appropriate horseradish peroxidase-conjugated secondary antibodies (1:10000, Promega), followed by chemiluminescence detection with an ECL Prime Western blotting detection kit (GE Healthcare). At least three replicates of each sample were analyzed by using the ImageQuant LAS 4000 software (GE Healthcare).

### Determination of the percentage of ERCs

Total cellular DNA was obtained from saturated YPD cultures as previously described. Approximately 10 μg of undigested genomic DNA were resolved by gel electrophoresis at a low voltage (0.7% agarose, 1.5 V/cm for 40 h) in TAE buffer (40 mM Tris, 20 mM acetate, 1 mM EDTA, pH 8). Gel was soaked in 0.25 M HCl for 10 min and DNA was transferred to a positively charged nylon membrane under alkaline conditions (0.4 M NaOH, 1 M NaCl) for at least 8 h. Membranes were then neutralized (0.5 M Tris-Cl pH 7.2, 1 M NaCl) for 15 min and successively hybridized with ^33^P-labelled ACT1 and rDNA probes. Probes were generated from a template that was PCR-amplified from genomic DNA and random-primed labeled with the High Prime kit (Sigma). After hybridization (24 h at 65°C in 0.5 M phosphate buffer, 1 mM EDTA, 7% SDS), blots were washed with washing solution I (1x SSC, 0.1% SDS) and washing solution II (0.5x SSC, 0.1% SDS) at 65°C. ERCs and *ACT1* levels were defined with a phosphorimaging system and quantification was done using the ImageJ software. The ERC and genomic rDNA values were normalized against the *ACT1* values.

## Supporting information

S1 Fig The number of rDNA repeats changes in cells with a different volume. A) The number of rDNA repeats per genome in different strains is constant, except for cell size haploid mutants. A t-test was used for statistical significance: ***: p-value < 0.0005; the other samples were not statistically different. B) The average cell volume (in arbitrary units) increases with the rDNA repeat copy number in a list of mutant strains from [29]. This graph is complementary to Fig 2B, but used the cell volumes from [34]. C) Cell volumes in the different strains used in this study. A t-test was used for statistical significance: ***: p-value < 0.0005; **: p-value < 0.005. D) A scheme of the rDNA locus and the probes used for qPCR is shown. Strain NOY408-1b (“1b”, see S1 Table), with a known rDNA copy number (150 repeats, see [34]), was used as an internal control.
(PDF)

S2 Fig Extrachromosomal rDNA circles (ERCs) analysis. The DNA from different strains was isolated and quantified. About 10 μg of DNA were electrophoresed in 0.7% agarose gel in TAE buffer at 1.5 v/cm for 40 h. A sample of BY4741 (wt) DNA was digested with *Bam*HI and was used as a size marker (left lane). DNA was transferred by alkaline Southern blot and successively hybridized with ACT1 and rDNA (18S) probes. The signal from ACT1 hybridization was used for normalization between samples to obtain the relative genomic rDNA repeats number (see Fig 2D). The sum of all the ERC bands, which corresponded to different repeat copy number ERCs [30,31], was related to the rDNA genomic band. The *sir2* strain was used as a control because it was described to have a high proportion of ERCs [47]. The size of the *Bam*HI *ACT1* band (16.5 kb) is shown. As the rDNA repeat has no *Bam*HI cutting site, it shows a band for it in the genomic DNA position.
(PDF)

S1 Table Yeast strains used in this study. (PDF)

S2 Table Oligonucleotides used in this study. (PDF)

S1 Appendix Integral Feedback Mathematical Model. (PDF)
