# Supplementary figures and images for: Cell volume homeostatically controls the rDNA repeat copy number and rRNA synthesis rate in yeast
Source: PLoS Genet. 2021 Apr 7;17(4):e1009520. doi: 10.1371/journal.pgen.1009520 (PMC8055003; doi:10.1371/journal.pgen.1009520)

# Supplementary Figure 1

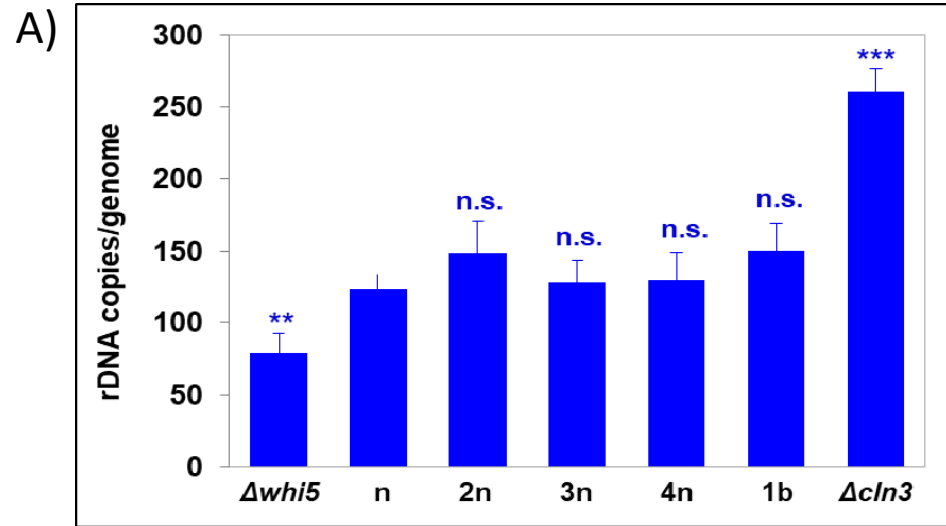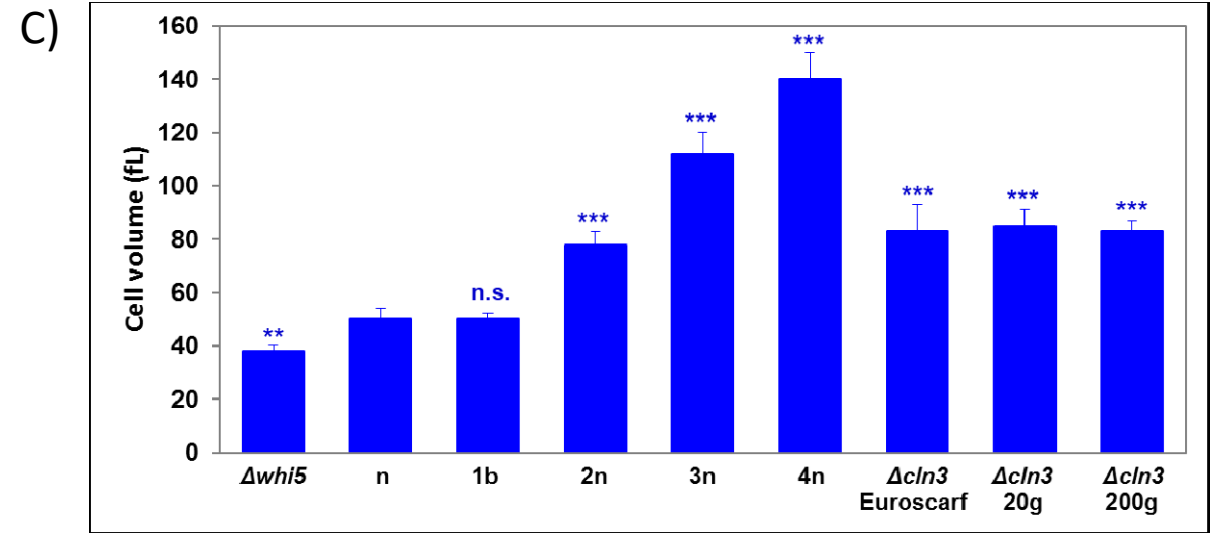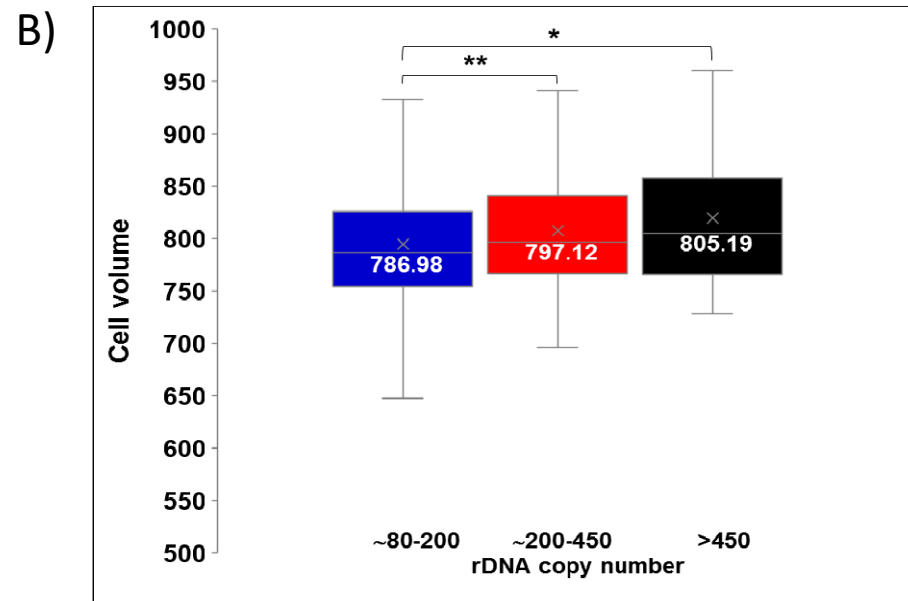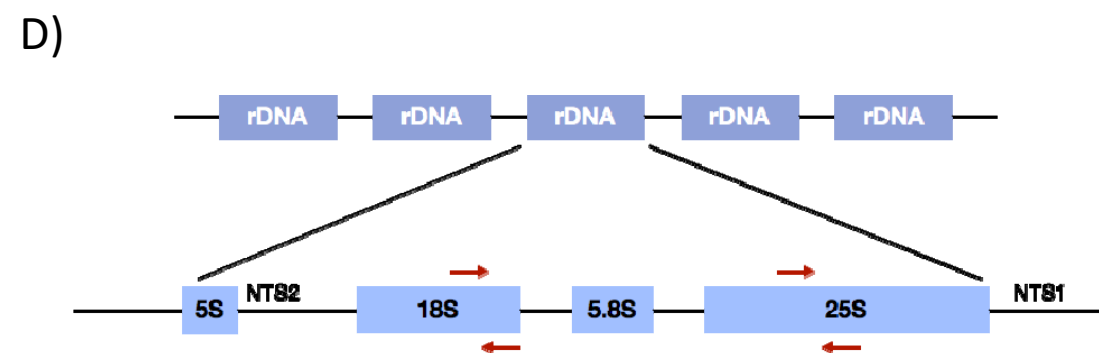

Supplement: S1 Fig — A) The number of rDNA repeats per genome in different strains is constant, except for cell size haploid mutants. A t-test was used for statistical significance: ***: p-value < 0.0005; the other samples were not statistically different. B) The average cell volume (in arbitrary units) increases with the rDNA repeat copy number in a list of mutant strains from [29]. This graph is complementary to Fig 2B, but used the cell volumes from [34]. C) Cell volumes in the different strains used in this study. A t-test was used for statistical significance: ***: p-value < 0.0005; **: p-value < 0.005. D) A scheme of the rDNA locus and the probes used for qPCR is shown. Strain NOY408-1b (“1b”, see S1 Table), with a known rDNA copy number (150 repeats, see [34]), was used as an internal control. (PDF) [file pgen.1009520.s001.pdf]

## Supplementary Figure 2

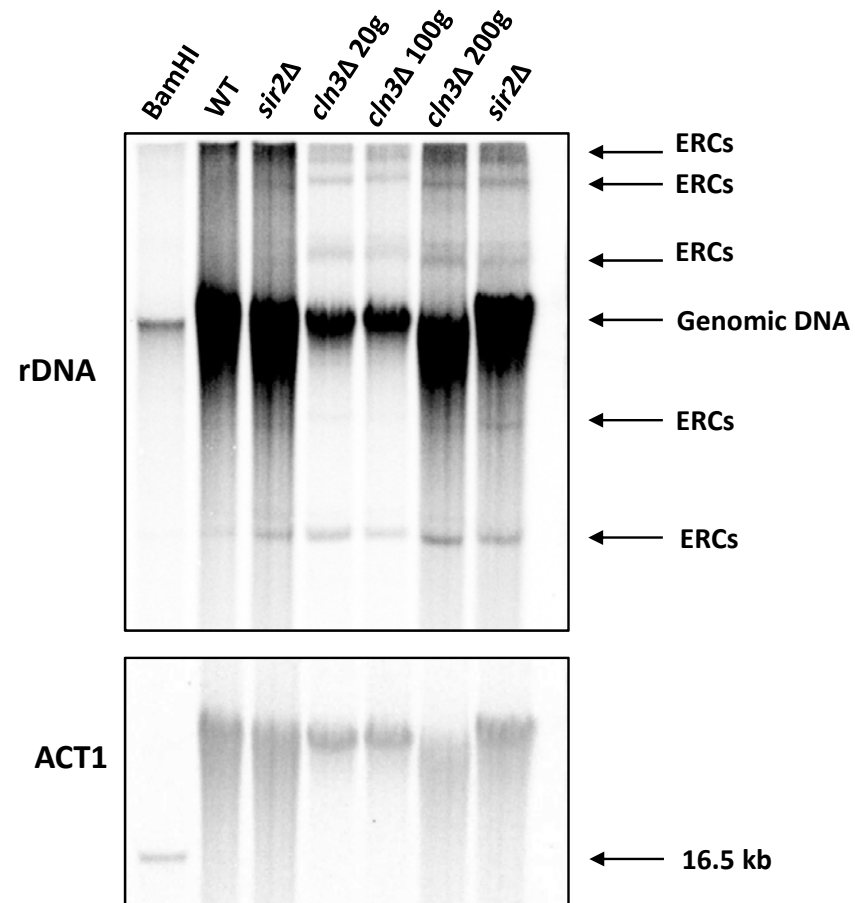

Supplement: S2 Fig — The DNA from different strains was isolated and quantified. About 10 μg of DNA were electrophoresed in 0.7% agarose gel in TAE buffer at 1.5 v/cm for 40 h. A sample of BY4741 (wt) DNA was digested with BamHI and was used as a size marker (left lane). DNA was transferred by alkaline Southern blot and successively hybridized with ACT1 and rDNA (18S) probes. The signal from ACT1 hybridization was used for normalization between samples to obtain the relative genomic rDNA repeats number (see Fig 2D). The sum of all the ERC bands, which corresponded to different repeat copy number ERCs [30,31], was related to the rDNA genomic band. The sir2 strain was used as a control because it was described to have a high proportion of ERCs [47]. The size of the BamHI ACT1 band (16.5 kb) is shown. As the rDNA repeat has no BamHI cutting site, it shows a band for it in the genomic DNA position. (PDF) [file pgen.1009520.s002.pdf]
