# Supplementary material for: Cell volume homeostatically controls the rDNA repeat copy number and rRNA synthesis rate in yeast
Source: PLoS Genet. 2021 Apr 7;17(4):e1009520. doi: 10.1371/journal.pgen.1009520 (PMC8055003; doi:10.1371/journal.pgen.1009520)
Supplement: S1 Table — (PDF) [file pgen.1009520.s003.pdf]

Strain	Genotype	Source	Ploidy	Doubling time (h)	Volume (fL)	Relative Volume
**BY4741 (n)**	*MAT a, leu2∆0, his3∆1, met15∆0, ura3∆0*	Euroscarf	n	1.7	50 ± 4	1
**PY4993 (2n)**	*MAT a/α; ura3∆0/ura3∆0, leu2∆0/leu2∆0, his3∆1/his3∆1, met15∆0/MET15, LYS2/lys2∆0*	Pellman Lab	2n	1.7	78 ± 5	1.57
**PY4997 (3n)**	*MAT a/a/α; ura3∆0/ura3∆0/ura3∆0, leu2∆0/leu2∆0/leu2∆0, his3∆1/his3∆1/his3∆1, met15∆0/MET15/MET15/, lys2∆0/lys2∆0/LYS2*	Pellman Lab	3n	1.7	112 ± 8	2.26
**PY4996 (4n)**	*MAT a/a/α/α;;ura3∆0/ura3∆0/ura3∆0/ura3∆0, leu2∆0/leu2∆0/leu2∆0/leu2∆0, his3∆1/his3∆1/his3∆1/his3∆1, met15∆0/met15∆0/MET15/MET15/, lys2∆0/lys2∆0/LYS2/LYS2*	Pellman Lab	4n	1.7	140 ± 10	2.83
**JCY0704**	BY4741 *whi5::LEU2*	JC Igual Lab	n	1.9	38 ± 3	0.76
**BQS2006 (Euroscarf *cln3Δ*)**	Haploid strain derived from Y30366 diploid (Euroscarf). *MAT?; ura3Δ, leu2Δ, HIS3, MET15, lys2Δ, cln3::KanMX4*	our lab	n	1.9	83 ± 10	1.68
**BQS278**	BY4741 *sir2::KanMX4*	our lab	n	nd	nd	nd
**NOY408-1b**	*MAT a ade2-1 ura3-1 his3-11 trp1-1 leu2-3, 112 can1-100 pNOY102*	Kobayasi Lab	n	2	43.7 ± 9	1.02*

***Relative volume values were obtained by considering BY4741 volume when growing in YPGal. Rest of volumes obtained from growth in YPD exponential phase. n.d. not determined**

Supplementary Table 1
