# Supplementary material for: Cell volume homeostatically controls the rDNA repeat copy number and rRNA synthesis rate in yeast
Source: PLoS Genet. 2021 Apr 7;17(4):e1009520. doi: 10.1371/journal.pgen.1009520 (PMC8055003; doi:10.1371/journal.pgen.1009520)
Supplement: S2 Table — (PDF) [file pgen.1009520.s004.pdf]

Oligonucleotides used in this study

Name	5'-3' Sequence
**RT-qPCR assays**	
ACT1-Forward	TCGTTCCAATTTACGCTGGTT
ACT1-Reverse	CGGCCAAATCGATTCTCAA
SIR2-Forward	ATTTATGCACGACCCCTCTG
SIR2-Reverse	TGGCACTGCACCAGTTTATC
UAF30-Forward	GAATTGACGAGGACCGAAGTAG
UAF30-Reverse	CGTCGCACAGTATTTCTTTCTTG
5.8S-Forward	GTTGCGGCCATATCTACCAG
5.8S-Reverse	AGCACCTGAGTTTCGCGTAT
18S-Forward	CATGGCCGTTCTTAGTTGGT
18S-Reverse	ATTGCCTCAAACTTCCATCG
25S-Forward	AGAGCCAATCCTTATCCCG
25S-Reverse	GGAAGCTCCGTTTCAAAGG
**Gene disruption**	
CLN3_KAN-Forward	CTTTTACTCTCGTTCAAGACACTGATTTGATACGCTTTCTGTACGC GTACGCTGCAGGTCGAC
CLN3_KAN-Reverse	AAATTTTAATTTATTTGTTGTTAAATGCATTTTTTTTTTGTCGTTATC GATGAATTCGAGCTCG
**Generation of Southern probes**	
ACT1-Forward	GTATTTTCACGCCGATAGATG
ACT1-Reverse	TTGGTCTACCGACGATAGATG
18S-Forward	CGAATTCCAGCTGACCACCATGTCAATGTCTTCGGA
18S-Reverse	AGGACTCAAGGTTAGCC

Supplementary Table 2
