# Supplementary material for: Cell volume homeostatically controls the rDNA repeat copy number and rRNA synthesis rate in yeast
Source: PLoS Genet. 2021 Apr 7;17(4):e1009520. doi: 10.1371/journal.pgen.1009520 (PMC8055003; doi:10.1371/journal.pgen.1009520)
Supplement: S1 Appendix — (PDF) [file pgen.1009520.s005.pdf]

Appendix: Integral Feedback

Consider the following system of chemical reactions which correspond to UAF molecules binding/unbinding to rDNA

$$UAF_{free} + rDNA \xrightarrow{k_{on}} UAF_{bound},$$
$$UAF_{bound} \xrightarrow{k_{off}} UAF_{free} + rDNA.$$

We assume that the total number of UAF molecules inside the cell scales with volume, and is given by cV, where V is the cell volume, and c is the UAF concentration. Then the number of bound UAF molecules bound to rDNA is given by

$$\frac{ck_{on}mV}{k_{off}V + k_{on}m + k_{on}cV} \tag{1}$$

where m is the number of rDNA repeats. In the limit of strong binding of UAF to rDNA ($k_{off} \ll k_{on}c$), the concentration of free UAF molecules

$$\frac{c^2}{m/V + c} \tag{2}$$

increases with increasing V. We assume that the production of SIR2 is a monotonically decreasing function of $\frac{c^2}{m/V+c}$ and given by $f\left(\frac{c^2}{m/V+c}\right)$. Let $x(t)$ denote the level of SIR2, then it evolves as per the differential equation

$$\frac{dx}{dt} = f\left(\frac{c^2}{m/V + c}\right) - \gamma_x x \tag{3}$$

where γ_x is the SIR2 degradation rate and time t can be interpreted in terms of number of generations. The number of rDNA repeats $m(t)$ follows the

differential equation

$$\frac{dm}{dt} = mg(x) - \gamma_m m \tag{4}$$

where γ_m and $g(x)$ are rates of deletion and expansion of rDNA repeats, respectively. A key feature here is that $g(x)$ is a monotonically decreasing function of SIR2 levels. The mathematical model given by (3)-(4) has been referred to in literature as *integral feedback control* (Aoki et al, Nature 2019) and it ensures that the the concentration of rDNA repeats is robustly maintained at a set point. To see this, consider equation (4) at steady-state, which gives the equilibrium SIR2 levels as

$$\bar{x} = g^{-1}(\gamma_m) \tag{5}$$

where g^{-1} denotes the inverse transformation of g. Now using (3) we can see that the steady-state rDNA repeat concentration $\bar{m}/V$ should satisfy

$$\frac{c^2}{\frac{\bar{m}}{V} + c} = f^{-1}(\gamma_x g^{-1}(\gamma_m)) \tag{6}$$

implying the setpoint

$$\frac{\bar{m}}{V} = \frac{c^2}{f(\gamma_x g^{-1}(\gamma_m))} - c. \tag{7}$$

In essence, an increase in volume results in an increase in the concentration of free UAF molecule as per (2). This causes the SIR2 production rate, and its levels x to decrease, which in turn increases the rate of expansion

of rDNA repeats $g(x)$. As the number of rDNA repeats increases, it brings down the concentration of free UAF, increases SIR2 levels, and decreases $g(x)$. Finally, homeostasis is restored when the number of rDNA increases such that its concentration is back to its set point (7).

For the simulation in Fig. 5, we assume a simple inverse-form for the functions f and g

$$f(x) = \frac{k_1}{x}, \quad g(x) = \frac{k_2}{x} \tag{8}$$

which leads to the model

$$\frac{dx}{dt} = k_1 \left(\frac{m}{c^2 V} + \frac{1}{c} \right) - \gamma_x x, \quad \frac{dm}{dt} = m \left(\frac{k_2}{x} - \gamma_m \right) \tag{9}$$

with the steady-state solution

$$\bar{x} = \frac{k_2}{\gamma_m}, \quad \bar{m} = V \left(\frac{k_2 \gamma_x c^2}{k_1 \gamma_m} - c \right). \tag{10}$$

We next define two dimensionless variables by normalizing $x(t)$ and $m(t)$ by their steady-state values in WT that is assumed to have a volume $V = 1\ a.u.$

$$\hat{x} = \frac{x}{\bar{x}}, \quad \hat{m} = \frac{m}{\frac{k_2 \gamma_x c^2}{k_1 \gamma_m} - c}. \tag{11}$$

Rewriting model (10) in terms of these dimensionless variables yields

$$\frac{d\hat{x}}{dt} = \frac{k_1 \gamma_m}{k_2 c} \left(\frac{\hat{m} \left(\frac{k_2 \gamma_x c}{k_1 \gamma_m} - 1 \right)}{V} + 1 \right) - \gamma_x \hat{x}, \quad \frac{d\hat{m}}{dt} = \gamma_m \hat{m} \left(\frac{1}{\hat{x}} - 1 \right). \tag{12}$$

For the simulation in Fig. 5, we run the above model assuming a two-fold increase in volume to $V = 2\ a.u.$,

$$\frac{k_1 \gamma_m}{k_2 c} = 1 \tag{13}$$

and $\gamma_x = 2$ per generation. As expected, SIR2 shows a rapid dip and increases back to the original level as in the WT, while the number of rDNA repeats shows a two-fold increase over generations keeping its concentration the same as in the WT.
